# Inhibition of Akt Attenuates RPO-Induced Cardioprotection

**DOI:** 10.1155/2012/392457

**Published:** 2012-10-03

**Authors:** Emma Katengua-Thamahane, Anna-Mart Engelbrecht, Adriaan J. Esterhuyse, Jacques Van Rooyen

**Affiliations:** ^1^Experimental Anti-oxidant Research Division, Department of Biomedical Sciences, Faculty of Health and Wellness Sciences, Cape Peninsula University of Technology, Symphony Road, Western Cape, Bellville 7535, South Africa; ^2^Department of Physiological Sciences, University of Stellenbosch, Stellenbosch 7600, South Africa

## Abstract

Previous studies have shown that red palm oil (RPO) supplementation protected rat hearts against ischaemia-reperfusion injury. Evidence from these studies suggested that Akt may be partly responsible for the observed protection. The aim of the current study was therefore to prove or refute the involvement of Akt in the RPO-induced cardioprotection by administration of a specific Akt inhibitor (A6730). Male Wistar rats were randomly divided into 2 groups: a control group receiving standard rat chow and an experimental group receiving standard rat chow plus 2 mL RPO for six weeks. Hearts were excised and mounted on the Langendorff perfusion system. Functional recovery was documented. A different set of hearts were freeze-clamped to assess total and phosphorylation status of Akt. Another set of hearts were subjected to the same perfusion conditions with addition of A6730. Hearts from this protocol were freeze-clamped and assessed for total and phospho-Akt. RPO improved functional recovery which was associated with increased phosphorylation of Akt on Ser473 and Thr308 residues. Blockade of Akt phosphorylation caused poor functional recovery. For the first time, these results prove that Akt plays an important role in the RPO-induced cardioprotection.

## 1. Introduction

Coronary heart disease (CHD) represents a major challenge to the health care systems in modern society and is the leading cause of death in the world [1]. The aetiology and pathophysiology of CHD is multifactorial. It is characterized by abnormal lipid metabolism, abnormal calcium homeostasis, endothelial dysfunction, hyperglycaemia, and an increased production of reactive oxygen species (ROS) [2]. Scientific evidence indicates that increased production of ROS represents a significant risk factor in the pathogenesis of CHD [3]. The role of oxidative stress has been well established in the pathogenesis of cardiovascular disease [4]. Plant-based foods and beverages have been shown to have beneficial effect on cardiovascular health [5, 6]. 

Consumption of foodstuffs which are rich in natural antioxidants could hold the key to reducing morbidity and mortality associated with diseases such as CHD, where oxidative stress is known to play an important role. Red palm oil (RPO) is refined edible oil obtained from crude palm oil through a special process of decalcification and deodorization using molecular distillation [7]. RPO is rich in natural vitamin E, containing 600–1000 ppm of tocopherols and tocotrienols [8]. Tocopherols and tocotrienols are fat soluble vitamin E isomers and are major antioxidants found in vegetable oils [9, 10]. The other antioxidants of physiological importance contained in red palm oil include carotenoids, squalene, and Co enzyme Q_10_ [11, 12]. RPO consists of almost equal amounts of saturated and unsaturated fatty acids [13]. The major saturated fatty acid is palmitic fatty acid, whilst oleic acid and linoleic acid are the major unsaturated fatty acids [14].

Experimental studies show that the cardioprotective effects of RPO may not only be due to the high antioxidant content found in the oil but could be mediated by the ability of RPO to modulate signalling events during ischaemia and reperfusion [6, 15–17]. The cardioprotective effects of the tocotrienol rich fraction have also been attributed to the ability of palm tocotrienol to modulate the Akt signalling, thus generating a survival signal during reperfusion [18]. Other natural substances such as *Ginkgo biloba* extract have been reported to offer cardioprotection against ischaemic insult in the isolated perfused rat heart model. In this regard Tosaki et al. [19] demonstrated that *Ginkgo biloba* extract improved contractile function in hearts subjected to ischaemia in a working heart model by reducing the formation of free radicals. In another study, Tosaki et al., 1996 reported that *Ginkgo biloba* extract improved cardiac function after ischaemia in both nonpreconditioned and preconditioned nondiabetic and diabetic rats [19, 20].

Previous studies have implicated Akt as a possible mechanism of protection against ischaemia-reperfusion injury in the cardioprotection mediated by RPO [15, 17]. Engelbrecht et al., 2006 reported that RPO supplementation improved post-ischaemic functional recovery. The improved functional recovery was associated with increased phosphorylation of Akt. The same group demonstrated that inhibition of PI-3 kinase attenuated postischaemic functional recovery in RPO supplemented hearts. Engelbrecht et al., 2009 concluded from their study that the beneficial effects of RPO are partially mediated via the PI-3/Akt signalling pathway. Their results showed that both Akt and its associated downstream targets were phosphorylated by RPO supplementation. These findings strongly suggested that Akt may play an important role in the RPO-induced cardioprotection. However, this evidence was circumstantial since PI-3 kinase has several downstream targets other than Akt. Specific inhibition of Akt will allow us to elucidate the importance of Akt on post-ischaemic functional recovery in RPO supplemented animals. In order to ascertain RPO induced Akt cardioprotection, we employed the Langendorff perfused heart model with the aid of specific Akt inhibitor (A6730). Instead of using wortmannin or LY294002 which are normally used to inhibit Akt via the PI3 K, we used A6730 which has been shown to specifically inhibit Akt phosphorylation. 

The aims of this study were

1. to investigate the effect of dietary RPO supplementation on functional recovery and on dual phosphorylation of Akt;
2. to either prove or refute the significance of Akt phosphorylation in RPO-induced cardioprotection by administration of a specific Akt inhibitor (A6730).

## 2. Materials and Methods

All animals used in this study received humane care in accordance with the Principle of Laboratory Animal Care of the National Society of Medical Research and the Guide for the Care and Use of Laboratory animals of the National Academy of Sciences (National Institutes of Health Publications no. 80-23, revised 1978). The rats had free access to water and food. They were housed in an animal house at a constant temperature of 27°C and they were exposed to a twelve-hour artificial day-night cycle. The ethical clearance for this study was granted by the Health and Applied Sciences Research Ethics Committee of the Cape Peninsula University of Technology (Ref: CPUT/HAS-REC 0019). 

### 2.1. Experimental Model

Male Wistar rats weighing 120–150 g were randomly divided into 2 groups. A control group receiving standard rat chow for six weeks and experimental group receiving standard rat chow plus 2 mL RPO for six weeks. RPO was mixed with a single pellet of the rat chow every day, and then they were only fed the rest of the daily rat chow after they consumed the pellet with the RPO. The rats in the control group consumed an average of 25 g standard rat chow/day, while the experimental group consumed the same amount of food plus additional 2 mL RPO. Similar previous studies have made use of the 2 mL RPO/diet. The rationale for using 2 mL RPO was based on earlier studies by Serbinova and coworkers who used 0.2 mL of the RPO baking fat [21]. The RPO product used in this study is 10 times less concentrated than the RPO baking fat; hence, 2 mL was used in this particular study. The approximate energy and nutritional composition of the

### 2.2. Heart Perfusion Protocol (Figure 1)

The study was divided into two perfusion protocols. In the first protocol, hearts from rats weighing 300–350 were anaesthetized with an intraperitoneal injection of 2 mg/kg intraval sodium (sodium pentobarbital), hearts were rapidly excised and placed in ice-cold krebs-Henseleit buffer, hearts were then transferred to the Langendorff perfusion apparatus and perfused according to the protocol of Engelbrecht et al., 2009 [17].

A balloon, made from transparent sandwich wrap film, was inserted into the left ventricle through the opening of the left atrium. The balloon was connected to a power lab system (AD Instruments Pty Ltd., Castle Hill, Australia) on a computer. After insertion, the balloon was inflated to 2 mmHg, and the contraction force of the heart against the balloon caused water displacement that was converted to pressure. The systolic and diastolic pressures as well as the heart rate were documented on the computer. LVDevP and RPP were used to quantify myocardial function. LVDevP is defined as the difference between the measured systolic pressure and the set diastolic pressure. RPP is calculated by multiplying LVDevP and heart rate. 

**Figure 1:**
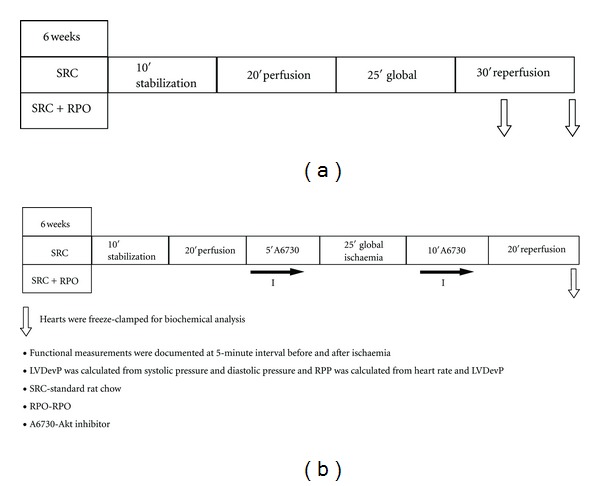
(a) Study design, perfusion protocol 1. (b) Study design, Perfusion protocol 2 and time points for biochemical samples were collected.

In the second perfusion protocol hearts were stabilized for 10 minutes and perfused for 20 minutes with normal Krebs-Henseleit buffer. Hearts were then perfused with 2.5 *μ*M Akt inhibitor (A6730) dissolved in 0.025% dimethylsulfoxide (DMSO) as a vehicle for the last 5 minutes of the perfusion period before being subjected to 25 minutes of total global ischaemia Figure 1(b). During the first 10 minutes of reperfusion, hearts were again reperfused with the Akt inhibitor for the first 10 minutes of reperfusion before reverting back to the Krebs-Henseleit buffer for the rest of the reperfusion period. Barnett et al. (2005) showed that the IC_50_ values for Akt1 and Akt2 were 2.7 *μ*M and 21 *μ*M, respectively [22]. However, in our model the concentration of 2.5 *μ*M significantly inhibited Akt phosphorylation after 30 minutes of reperfusion.

### 2.3. Akt Analysis

For analysis of total and phospho-Akt hearts from all groups were freeze-clamped 10 minutes and 30 minutes into reperfusion. Cardiac proteins were extracted with a lysis buffer containing (in mM): Tris 20, p-nitrophenylphosphate 20, EGTA 1, NaF 50, sodium orthovanadate 0.1, phenylmethyl sulfonyl fluoride (PMSF) 1 m dithiothreitol (DTT) 1, and aprotinin 10 *μ*g/mL. The tissue lysates were diluted in Laemmli sample buffer, boiled for 5 minutes and 50 *μ*g proteins per lane were separated by 10% PAGE-SDS gel electrophoresis. The lysate protein content was determined using the Bradford technique [23]. Proteins were transferred to a PVDF membrane (Immobilon P, Millipore). These membranes were routinely stained with Ponceau Red for visualization of proteins. Nonspecific binding sites on the membranes were blocked with 5% fat-free milk in Tris-buffered saline−0.1% Tween 20 (TBST) and then incubated with the primary antibodies that recognize Akt (Ser473 and Thr308) and total Akt. Membranes were subsequently washed with large volumes of TBST (5 × 3 minutes) and incubated with the secondary antibody conjugated with alkaline-phosphatase for one hour with continuous shaking at room temperature. After thorough washing with TBS-T, membranes were covered with a chromogenic substrate (Protein Detector BCIP/NBT Western Blotting Kit, invitrogen) and subsequently densitometrically analysed. 

### 2.4. Data Analysis

Results are expressed as mean ± standard error of the mean (SEM). Differences between the groups were determined using an unpaired Student's *t*-test and to compare differences in multiple groups, a one-way ANOVA with a Benferroni Multiple comparison as a post hoc test was used. *P* < 0.05 was considered to be statistically significant.

## 3. Results

Preischaemic LVDevP (mmHg) baseline values and post-ischemic LVDevP absolute values for controls and experimental groups are shown in Table 1. LVDevP recoveries (%) are shown in Table 2. Dietary RPO supplementation significantly improved post-ischaemic functional recovery as reflected by increased LVDevP recovery (%) in experimental animal compared to controls at specific reperfusion time points, Table 2. The LVDevP recovery (%) in RPO hearts was significantly improved from 15 to 30 minutes of reperfusion; RPO versus control at 15 minutes (97.25 ± 3.57% versus 84.40 ± 4.24%; *P* < 0.05), at 20 minutes (96.23 ± 3.94% versus 79.36 ± 3.19%; *P* < 0.01), at 25 minutes (96.23 ± 3.94% versus 79.36 ± 30.190%; *P* < 0.01), and at 30 minutes (86.70 ± 11% versus 72.21 ± 2.71%; *P* < 0.01). 

**Figure 2:**
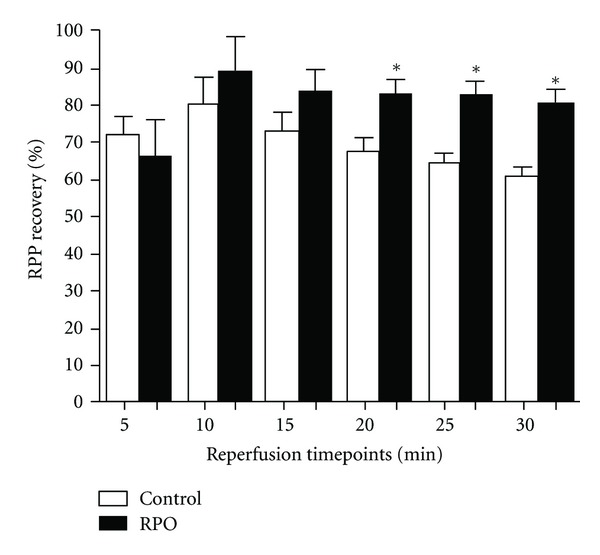
The Effect of RPO on RPP recovery (%). Results are expressed as mean ± SEM, (*P < 0.05 for indicated groups). (Control, *n* = 7 and RPO, *n* = 7) RPO-Red palm.

Post-ischaemic RPP (mmHg/min) recoveries are expressed as a percentage of pre-ischaemic baseline values. RPO significantly improved RPP recovery (%) compared to controls. Significant differences between RPO hearts and controls were observed from 20 minutes to 30 minutes of reperfusion; RPO versus control at 20 minutes reperfusion (87.58 ± 4.05% versus 73.05 ± 3.26%; *P* < 0.05), 25 minutes (87.06 ± 3.41% versus 73.78 ± 4.42%; *P* < 0.05) and 30 minutes (81.27 ± 4.58% versus 69.22 ± 3.37%; *P* < 0.05), Figure 2.

### 3.1. Effect of RPO on Total and Phosphorylated Akt on Ser473 and Thr308, (Figures 3(a), 3(b), and 3(c))

**Figure 3:**
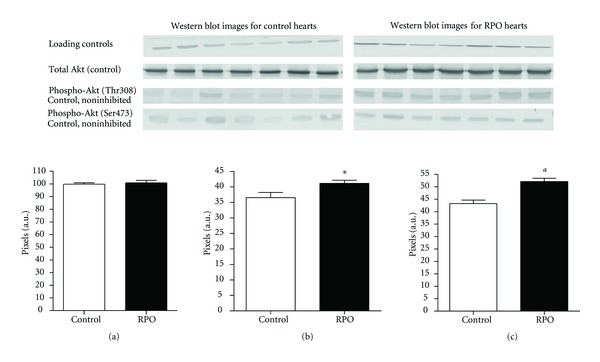
(a) The effect of RPO on total Akt during reperfusion. (b) The effect of RPO on phospho-Akt (Ser473) during reperfusion. (c) The effect of RPO on phospho-Akt (Thr308) during reperfusion.

There was no significant difference observed in total Akt between controls and the RPO supplemented group (Figure 3(a)). RPO caused dual phosphorylation of Akt on Ser473 and Thr308 residues (Figures 3(b) and 3(c)), RPO versus C (41.15 ± 1.010 pixels versus 36.54 ± 1.706 pixels; *P* < 0.05), Thr308 (52.13 ± 1.349 pixels versus 43.28 ± 1.413 pixels; *P* < 0.01), representative blot images attached, Figure 3. The results indicate that RPO supplementation significantly upregulated the phosphorylation of Akt during reperfusion which is in agreement with previous studies. 

### 3.2. Effect of RPO and Akt-1-1/2 Inhibitor (A6730) on LVDevP Recovery (%) (Figure 4)

RPO + A6730 hearts showed increased LVDevP compared to control + A6730 at 15 reperfusion, (76.36 ± 6.47% versus 57.72 ± 4.93%, *P* < 0.05). These results demonstrate that administration of A6730 decreased mechanical functional recovery to a lesser degree in RPO + A6730 hearts compared to control + A6730 hearts. RPO significantly improved LVDevP compared to controls at 20 minutes reperfusion (96.23 ± 3.70% versus 81.46 ± 2.4%, *P* < 0.05). Our results also show that at the same time point RPO significantly increased LVDevP recovery compared to control + A6730 hearts, (96.23 ± 3.70% versus 53.59 ± 2.41%; *P* < 0.01). The results show that at 25-minutes reperfusion, administration of the inhibitor significantly abrogated LVDevP in Akt-inhibited hearts compared to inhibitor-free hearts, RPO versus RPO + A6730 (96.23 ± 3.70% versus 64.24 ± 6.01%; *P* < 0.01) and for RPO versus control + A6730 (92.84 ± 4.00% versus 49.87 ± 2.47%; *P* < 0.01). The same trend results were also observed at 30-minute reperfusion,control versus control + A6730 (71.49 ± 2.74 versus 45.91 ± 2.96; *P* < 0.01), and for RPO versus RPO + A6730 (86.70 ± 4.11 versus 61.60 ± 6.15; *P* < 0.01). 

### 3.3. Effect of RPO and Akt-1-1/2 Inhibitor (A6730) on RPP (%), (Figure 5)

A6730 attenuated RPP recovery in control + A6730, but did not have the same effect on RPP in RPO + A6730 group. At 20-minute reperfusion the RPP recovery for RPO + A6730 versus control + A6730 was (90.35 ± 8.82% versus 65.78 ± 6.03% (*P* < 0.05), at 25 minutes RPO + A6730 versus control + A6730 (87.2 ± 8.96% versus 62.38 ± 3.91% (*P* < 0.05), and at 30 minutes RPO + A6730 versus control + A6730 (84.84 ± 9.78% versus 57.59 ± 3.52% (*P* < 0.05). The RPP recoveries in RPO hearts were increased compared to control + A6730 after 25 and 30 minutes of reperfusion, RPO versus control + A6730 group at 25-minute reperfusion (87.06 ± 3.82% versus 62.38 ± 3.919% (*P* < 0.05), and at 30 minutes RPO versus control + A6730 (81.27 ± 4.58% versus 57.59 ± 3.52% (*P* < 0.05).

### 3.4. Effect of RPO and A6730 on Akt Phosphorylation (Phospho-Akt (Ser473) and Phospho-Akt (Thr308) (Figure 6)

**Figure 6:**
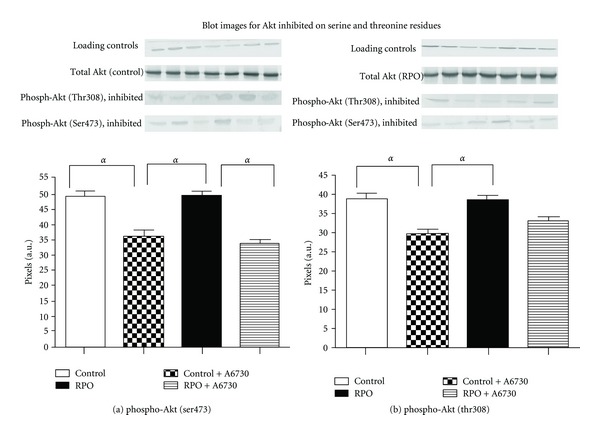
The effect of RPO and A6730 on Akt phosphorylation (Ser473 (a) and Thr308 (b) residues). Results are expressed as means ± SEM, *n* = 6-7/group (^*α*^ *P* < 0.01 for indicated groups).

Administration of A6730 significantly reduced phosphorylation of Akt, in Akt-inhibited hearts compared to the noninhibited hearts (Figure 6). Significant differences were observed in phosphorylation of Ser473 in the following groups: control versus control + A6730 (49.24 ± 1.59 pixels versus 36.97 ± 1.95 pixels RPO; *P* < 0.01), RPO versus control + A6730 (49.81 ± 1.33 pixels versus 36.97 ± 1.95 pixels; *P* < 0.01) and RPO versus RPO + A6730 (49.81 ± 1.33 pixels versus 34.18 ± 1.22 pixels; *P* < 0.01) (Figure 6(a)). Administration of A6730 also caused significant reduction in phosphorylation of Thr308 in the control group compared to control + A6730 after 30 minutes of reperfusion; control versus control + A6730 (38.92 ± 1.32 pixels versus 29.98 ± 0.84 pixels; *P* < 0.01), and also in the RPO group versus control A6730 (38.05 ± 1.71 versus 29.98 ± 0.84 pixels; *P* < 0.01), (Figure 6(b)), representative blot images attached, Figure 6.

## 4. Discussion 

We have demonstrated that dietary RPO supplementations offered significant post-ischaemic functional recovery. This was shown by a sustained post-ischaemic LVDevP recovery (%) from 15 minutes to 30 minutes of reperfusion. The results are in agreement with previous studies where RPO was reported to protect hearts from ischaemia-reperfusion injury in the working rat heart model [15]. We further demonstrated, for the first time, that RPO supplementation was associated with increased dual phosphorylation of Akt on Ser473 and Thr308 residues. Previous studies showed that the RPO-induced cardioprotection was associated with increased phosphorylation of Akt on Ser473 [15, 17]. Scientific evidence suggests that optimal activation of Akt requires phosphorylation on both Ser473 and Thr308 residues [24, 25]. Therefore, in this study we investigated the effect of RPO on these two key residues and found that RPO significantly increased phosphorylation of Akt on both Thr308 and Ser473 (Figures 3(b) and 3(c)). Our results indicate that Akt is a possible mechanism underlying RPO-induced cardioprotection in the isolated perfused rat heart model. 

Administration of A6730 caused significant reduction in contractile functional recovery, as evidenced by reduced LVDevP. A6730 partially reduced LVDevP recovery in the RPO + A6730, compared to control + A6730 Akt group, suggesting that there could be an alternative mechanism other than Akt phosphorylation by which RPO protected the heart against ischaemia-reperfusion injury. Similar results were reported by Engelbrecht and colleagues (2009), where RPO was found to offer better functional recovery in the presence of wortmannin [17]. 

The role of Akt as an important survival kinase in ischaemia-reperfusion injury has been well documented [26, 27]. It has been shown that Akt plays an important role in modulating myocardial contractility and intracellular calcium handling [28–30]. It is well known that the contractility of cardiac muscle is primarily dependent on the way the myocardial cells handle calcium ions. Therefore, we can argue that the improved mechanical functional recovery in RPO supplemented hearts could have been a direct effect of Akt on calcium ions. However, further studies will be needed to ascertain this hypothesis. Even though the role of Akt against ischaemia-reperfusion injury in rodents has been established in previous studies [26, 27], it still remains controversial if this survival kinase plays an important role in cardioprotection observed in larger animal species such as pigs [31]. However, there is credible, evidence to believe that Akt has cardioprotective effects against ischaemia-reperfusion injury [26, 27, 30, 32]. Akt mainly transmits mitogen signals towards its intracellular targets, but it has also been reported to promote cell survival upon oxidative insults [33]. Toth and coworkers reported that administration of free radical scavenging molecule protected myocardial cells from ischaemia/reperfusion injury by scavenging free radicals and moreover through its ability to upregulate the Akt pathway [32]. RPO is natural oil which is rich in antioxidants such as carotenes, tocopherols, and tocotrienols. The antioxidant vitamins in RPO have the potential to function as potent free radical scavengers. In our study, we do not attribute the cardioprotective effects of RPO to one particular component, but to all the active components of RPO which may synergistically support each other. It has been reported that palm tocotrienols mediated cardioprotection via their ability to maintain a balance between the prodeath and prosurvival signals [34]. These authors further demonstrated that tocotrienols inhibited the prodeath signals while increasing the activity of the Akt signaling. Evidence from previous studies indicates that the cardioprotective effect of RPO is not only due to its antioxidant content but also due to its ability to modulate signalling events during ischaemia-reperfusion [17, 18, 34]^.^ Bester and coworkers reported that the cardioprotective effect of RPO was associated with reduced myocardial infarct size and increased Akt phosphorylation [35]. Earlier studies by Bester et al. (2006) employed isocaloric diets with RPO supplementation in the isolated perfused rat heart model [36]. They reported that the difference in energy consumption was not responsible for the RPO cardioprotection observed, but rather a difference in the composition of the diets [36]. This also creates an opportunity to speculate about the role of the individual bioactives as potential protectors or the combinations of the bio-actives which synergistically may offer this protection. The combination of fat soluble antioxidants, such as carotenoids, tocopherols and tocotrienols, and specific fatty acids, such as oleic and linoleic acid, and the “minor” concentrations of components such as squalene and coenzyme Q10 will undoubtedly all play a role in the cellular events. Recently studies have shown that dietary RPO supplementation reduced myocardial infarct size after ischaemia-reperfusion injury [35, 37]. The reduction of infarction in RPO supplemented rats was also associated with a reduction of LDH in the coronary effluent showing that RPO protected against irreversible cardiomyocyte damage, which would ultimately lead to improved functional recovery. The current study used LVDevP as an end point of functional recovery which was recorded over 30 minutes. The question may be posed whether the observed protection was offered against reversible stunning or infarction. However, studies using a similar model where function was the focus have been published (Engelbrecht et al., 2006, Du Toit et al., 2001) [38, 39]. Myocardial stunning has been established as a manifestation of reperfusion injury [40, 41]. It may therefore suggests that the cardioprotection against the deleterious consequence of stunning will be translated to better functional recovery. Myocardial stunning is a complex phenomenon. The intension of this paper was not to investigate the effect of RPO on myocardial stunning but rather to investigate the importance of increased Akt phosphorylation on the functional recovery. 

## 5. Conclusion

We have for the first time shown that phosphorylation of Akt plays a significant role in the cardioprotection mediated by RPO. Administration of A6730 resulted only in partial attenuation of cardioprotection in RPO supplemented hearts, suggesting that other pathways could also be involved in this cardioprotection. Therefore, it can be concluded that Akt plays a partial, but significant role, in RPO-induced cardioprotection.

## Figures and Tables

**Figure 4:**
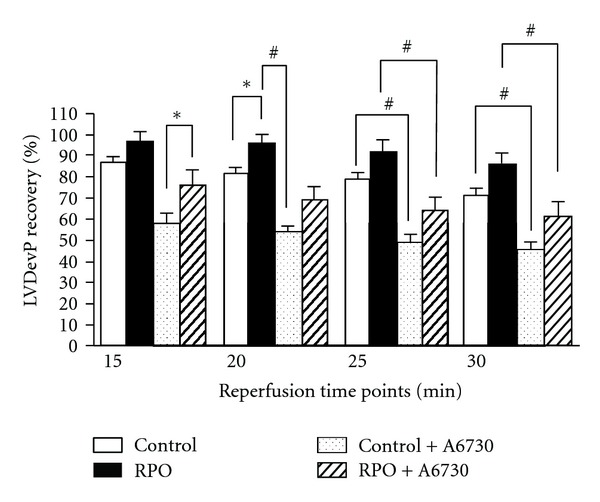
Effect of RPO and Akt-1-1/2 inhibitor (A6730) on LVDevP recovery (%). Results expressed as means ± SEM (*P < 0.05 and ^#^ *P* < 0.01 for indicated groups). Controls (*n* = 7), RPO (*n* = 7), control + A6730 (*n* = 5), and RPO + A6730 (*n* = 5).

**Figure 5:**
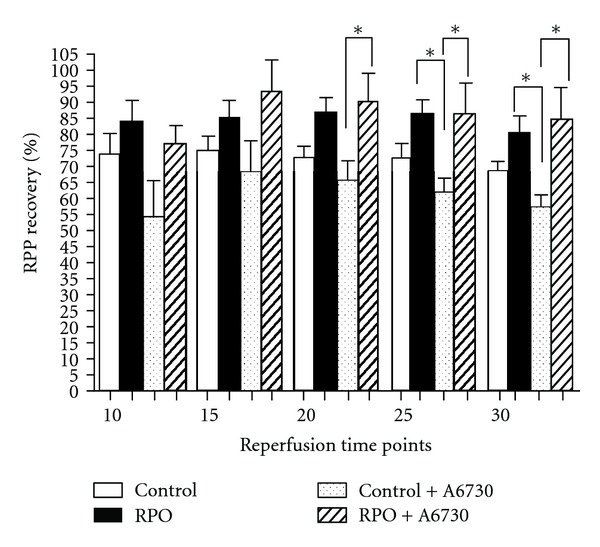
Effect of RPO and Akt-1-1/2 inhibitor (A6730) on RPP recovery (%). Results expressed as means ± SEM (*P < 0.05 for indicated groups). Controls (*n* = 7), RPO (*n* = 7), control + A6730 (*n* = 5), and RPO + A6730 (*n* = 5).

**Table 1:** LVDevP preischaemic baseline values and postischaemic LVDevP values for controls and RPO hearts at different reperfusion time points.

	Baseline LVDevP	10-minute reperfusion	15-minute reperfusion	20-minute reperfusion	25-minutes reperfusion	30-minutes reperfusion
Control	65.69 ± 3.06	67.82 ± 1.78	59.28 ± 5.37	55.17 ± 1.84	53.74 ± 2.10	48.33 ± 1.59
RPO	74.51 ± 4.67	75.28 ± 2.22	72.04 ± 3.91	72.16 ± 4.43	69.21 ± 5.38	64.55 ± 4.92

**Table 2:** Post-ischaemic % LVDevP recoveries for controls and RPO hearts at different reperfusion time points.

	10-minute reperfusion	15-minute reperfusion	20-minute reperfusion	25-minute reperfusion	30-minute reperfusion
Control	103.25 ± 3.74	87.25 ± 3.34	81.46 ± 2.41	79.32 ± 2.61	71.48 ± 2.74
RPO	101.03 ± 5.08	97.25 ± 3.34*	96.23 ± 4.3.46*	92.84 ± 3.75*	86.70 ± 3.85*
